# The Relation Between Complexity and Resilient Motor Performance and the Effects of Differential Learning

**DOI:** 10.3389/fnhum.2021.715375

**Published:** 2021-08-12

**Authors:** Ruud J. R. Den Hartigh, Sem Otten, Zuzanna M. Gruszczynska, Yannick Hill

**Affiliations:** ^1^Department of Psychology, Faculty of Behavioral and Social Sciences, University of Groningen, Groningen, Netherlands; ^2^Faculty of Medical Sciences, Center for Human Movement Sciences, University Medical Center Groningen/University of Groningen, Groningen, Netherlands; ^3^Institute of Sport and Sport Science, Heidelberg University, Heidelberg, Germany

**Keywords:** adaptability, complex systems, detrended fluctuation analysis, non-linear dynamics, pink noise

## Abstract

Complex systems typically demonstrate a mixture of regularity and flexibility in their behavior, which would make them adaptive. At the same time, adapting to perturbations is a core characteristic of resilience. The first aim of the current research was therefore to test the possible relation between complexity and resilient motor performance (i.e., performance while being perturbed). The second aim was to test whether complexity and resilient performance improve through differential learning. To address our aims, we designed two parallel experiments involving a motor task, in which participants moved a stick with their non-dominant hand along a slider. Participants could score points by moving a cursor as fast and accurately as possible between two boxes, positioned on the right- and left side of the screen in front of them. In a first session, we determined the complexity by analyzing the temporal structure of variation in the box-to-box movement intervals with a Detrended Fluctuation Analysis. Then, we introduced perturbations to the task: We altered the tracking speed of the cursor relative to the stick-movements briefly (i.e., 4 s) at intervals of 1 min (Experiment 1), or we induced a prolonged change of the tracking speed each minute (Experiment 2). Subsequently, participants had three sessions of either classical learning or differential learning. Participants in the classical learning condition were trained to perform the ideal movement pattern, whereas those in the differential learning condition had to perform additional and irrelevant movements. Finally, we conducted a posttest that was the same as the first session. In both experiments, results showed moderate positive correlations between complexity and points scored (i.e., box touches) in the perturbation-period of the first session. Across the two experiments, only differential learning led to a higher complexity index (i.e., more prominent patterns of pink noise) from baseline to post-test. Unexpectedly, the classical learning group improved more in their resilient performance than the differential learning group. Together, this research provides empirical support for the relation between complexity and resilience, and between complexity and differential learning in human motor performance, which should be examined further.

## Introduction

Human motor performance can be considered as inherently complex and dynamic. Whether it is about rhythmical finger tapping, leg movements, or more complicated movements on the sports field, an individual’s motor performance emerges out of simultaneous processes at different levels of the motor system, including cells, muscles, limbs, and the brain (e.g., Thelen et al., 1987; Beek et al., 1995; Kelso, 1995; Davids et al., 2014; Den Hartigh et al., 2015). Through these complex dynamics, the human motor system typically organizes itself around metastable states, meaning that its behavior demonstrates a mixture of order (regularity) and disorder (flexibility) (e.g., Kello et al., 2007). This optimal mixture is a hallmark of complexity and expresses itself in the fluctuations of individuals’ repeated movements, such as intervals between strides, arm movements or rowing strokes (e.g., Goldberger et al., 2002; Wijnants et al., 2009; Diniz et al., 2011; Marmelat and Delignières, 2012; Den Hartigh et al., 2015).

The characteristic pattern of variation in complex systems behavior is called fractal scaling, 1/f noise, or pink noise, showing high-frequency and low-amplitude fluctuations that are nested within low-frequency and high-amplitude fluctuations (e.g., Wijnants et al., 2012). This nested structure reflects the idea that a system consists of components across different structural levels and timescales that constantly interact. Accordingly, the current state of the system contains the “memory” of its previous states (i.e., long-range (fractal) correlations, see Diniz et al., 2011). In the past decades, accumulating research has demonstrated that diseased or lower-performing systems show deviations from a pink noise pattern, reflecting a loss of complexity. Early studies in this direction, for instance, focused on the temporal structure of heart beat intervals, in order to study the complexity of the cardiovascular system (e.g., Stanley et al., 1992; Peng et al., 1993). These studies typically showed prominent patterns of pink noise in the time series of heart beat intervals of healthy adults. On the other hand, a clear deviation from pink noise (e.g., random variation in intervals) appeared to be a signature of heart failure (see Goldberger et al., 2002). The methods to detect and quantify the temporal structure of heartbeat intervals could also be applied in research on motor performance. For example, a series of studies on human walking has demonstrated that stride interval time series of healthy young adults reveal patterns of pink noise, whereas the stride intervals of people with Huntington or Parkinson disease demonstrate patterns close to so-called white noise (e.g., Hausdorff et al., 1997b; Hausdorff, 2009). This white noise pattern means that more random variation in stride intervals is present, which suggests a worse dynamic organization of the motor system. Accordingly, researchers examining cyclic movements in sports found that skilled athletes demonstrate more prominent patterns of pink noise in their movements than their less-skilled counterparts (e.g., Den Hartigh et al., 2015 in rowing ergometer performance; Nourrit-Lucas et al., 2015 in ski simulator performance).

In line with the findings mentioned above, researchers have suggested that a loss of complexity likely accompanies an impaired ability to adapt to stress or perturbations (e.g., Lipsitz and Goldberger, 1992; Stergiou and Decker, 2011). This fits with the findings by Hausdorff et al. (1997a) that elderly people with higher propensities to fall show more prominent patterns of white noise in their stride intervals compared to elderly “non-fallers” and healthy young adults. It thereby appears that complexity provides the system with both robustness (maintaining proper functioning despite perturbations) and adaptability (adapting to changes or stressors in the environment) (Delignières and Marmelat, 2013; Almurad et al., 2018).

In the behavioral sciences, an individual’s ability to maintain robust functioning despite perturbations and to adapt to stressors is called *resilience* (e.g., Carver, 1998; Luthar et al., 2000; Masten, 2001; Smith et al., 2008; Pincus and Metten, 2010; Hill et al., 2018a). Although specific definitions of resilience show some variations, “each of these definitions encompasses complex adaptive change over time” (Pincus and Metten, 2010, p. 357). This complex adaptive change entails that a person is not governed by one fixed state, but can quickly transition between equally functional states if the environment demands adaptations (Kiefer et al., 2018). Proceeding from the idea that the concepts of complexity and resilience are probably related, our first aim was to test this relation in a motor task.

Another topic that has gained significant interest in the past two decades is how complexity, adaptation, and resilience may be improved in human behavior (e.g., Schöllhorn et al., 2012; Kiefer and Myer, 2015; Almurad et al., 2018; Hill et al., 2020b). According to various researchers, system complexity can be improved by exploiting variability (e.g., Van Emmerik and Van Wegen, 2000; Riley and Turvey, 2002; Davids et al., 2003; Stergiou and Decker, 2011; Schöllhorn et al., 2012; Seifert et al., 2013; Liu et al., 2015). Hence, a promising avenue is to apply interventions that take advantage of the important role of variability in finding functionally adaptive movement patterns. A particularly interesting application of this idea is differential learning, which is developed from the perspective of complex dynamical systems (e.g., Schöllhorn et al., 2006, 2009, 2010, 2012; Savelsbergh et al., 2010; Santos et al., 2018). In brief, a differential learning program introduces random noise to destabilize the system, and possibly strengthen the development of metastable states (e.g., Schöllhorn et al., 2009; Gray, 2020). This is in stark contrast with classical learning, which focuses on the development of an ideal movement pattern, deviations from which are considered detrimental to performance. Repetition and corrective feedback are therefore core ingredients of the latter approach. In a differential learning approach, on the other hand, performing various movements for the same task is stimulated without any corrective feedback while performing (Schöllhorn et al., 2006, 2009, 2012; Savelsbergh et al., 2010). Thereby, the learner actively explores a large range of possible motor solutions to a given task, which fosters behavioral adaptations (cf. Latash, 2012). Consequently, differential learning may lead to improved resilient motor performance (i.e., performance while being perturbed by stressors).

The benefits of differential learning have already been demonstrated in the domain of sports, such as soccer (Schöllhorn et al., 2006, 2012; Santos et al., 2018; Gaspar et al., 2019), speed skating (Savelsbergh et al., 2010), and baseball (Gray, 2020). For instance, Savelsbergh et al. (2010) trained novice speed skaters in their starting posture. The differential learning group began every start with a different posture and received no feedback on their performance. On the other hand, a classical learning group learned the starting posture as described in a skating handbook. A control group just engaged in regular speed skating lessons. The speed skating performance improved most for the participants in the differential learning group, suggesting that their system was better able to adapt to changing environmental constraints, resulting in better performance (Schöllhorn et al., 2006). In line with the possible benefits of differential learning, the second aim of the current study was to examine the effects of this type of learning on complexity and resilient motor performance.

In summary, complex systems tend to organize themselves around metastable states that would allow for adaptation when perturbations are imposed on their behavior. At the same time, the adaptation of a system to perturbations is a core characteristic of resilience (e.g., Pincus and Metten, 2010; Hosseini et al., 2016; Hill et al., 2018a,b). Hence, it is plausible that there is a link between the complexity of a system and its resilient performance. In order to improve complexity, and possibly resilience, differential learning may provide an interesting intervention that exploits the functional role of variability in system behavior. Following this rationale, our research question was: What is the relation between complexity and resilient motor performance, and can they be improved through differential learning? In order to answer this question, we developed a motor task that allowed us to (1) let participants perform repetitive (cyclical) movements, which are particularly useful for the analysis of complexity in time series (e.g., Wijnants et al., 2009, 2012), and (2) introduce perturbations while participants are performing the task. The specific task we used was a lateral movement task in which participants had to move a stick from left to right in order to move a cursor between two boxes on a screen in front of them. We conducted two parallel experiments with this task: one that included maintaining performance while being briefly perturbed at regular intervals, thereby emphasizing the robustness side of resilience, and one including prolonged perturbations that required changes to other performance states, thereby emphasizing the adaptability side. Across the two experiments, we expected a positive relation between complexity and resilient performance (*hypothesis 1*). Furthermore, in contrast to classical learning, we expected that differential learning leads to an improvement of complexity (i.e., more prominent patterns of pink noise in temporal performance fluctuations — *hypothesis 2*). Relatedly, we expected that, in contrast to classical learning, differential learning leads to better resilient motor performance (*hypothesis 3*). Finally, we explored whether the hypothesized effects differ depending on the type of stressors (brief vs. prolonged) introduced.

## Materials and Methods

### Participants

We recruited 82 participants who were living in The Netherlands. Most of these participants were first year psychology students recruited through a participant pool of the university. Thirty-nine participants started, and completed Experiment 1 (*M*_*age*_ = 20.54, *SD* = 2.06; 59% female), and 43 participants started the parallel Experiment 2, of which 40 actually completed it (*M*_*age*_ = 21.60, *SD* = 3.76; 58% female). In both experiments, participants were randomly allocated to either a classical learning condition or a differential learning condition. None of the participants had motor impairments, and all of them had normal or corrected-to-normal vision.

### Materials

**FIGURE 1:**
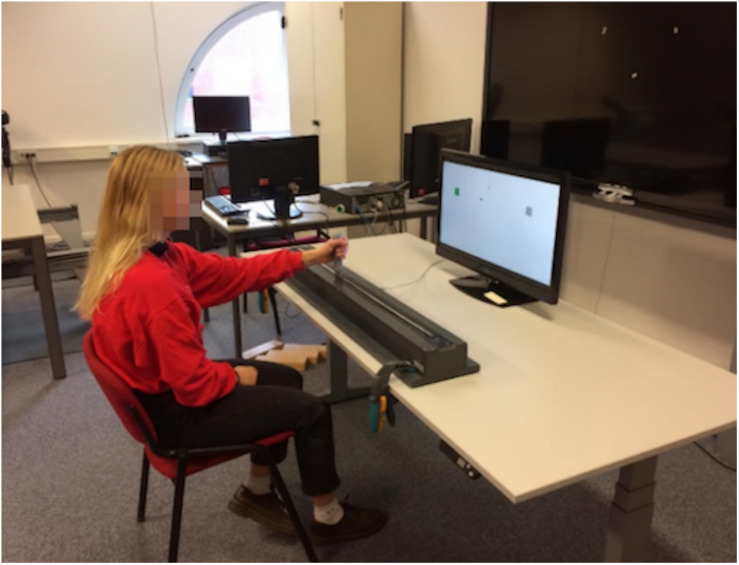
Illustration of the research setup.

The device used was designed for the purpose of the current research (see Figure 1). This device consisted of a meter-long rail, on which a stick was attached that could move along the entire length of the rail. The position of the stick was recorded with a frequency of 20Hz, and the movements were projected on a screen (Iiyama, 27 inch) in front of the participants. The screen was placed behind the device on a table with adjustable height. Participants could sit down on a non-adjustable chair without armrests.

The task consisted of a game in which participants had to move the cursor from left-to-right between two boxes on the screen. Each time they touched the box, the box turned green and participants won a point, but when overshooting the box turned red and a point was subtracted. The software we made for this research allowed us to manage different settings of the game, such as playing time, tracking speed of the cursor, size and placement of the squares, and graphic cues for the player (e.g., seeing elapsed time, points, or squares changing color when touched).

### Procedure

The protocol of the study was approved by the Ethics Committee Psychology of the University of Groningen. The parallel experiments consisted of three meetings, each lasting about 1 h, with a maximum of 5 days in between meetings to retain the influence of the preceding session. The first meeting included a baseline session and a first training session; the second meeting consisted of two training sessions; and the third meeting consisted of the last training session and a posttest. Participants had a 10 min break in between two sessions on the same day. Before the first meeting, we randomly allocated participants to either a classical learning condition or a differential learning condition. At the start of the first meeting, we explained the nature of the experiment and the task participants would have to carry out. We explained how the game had to be played (how to win points), and we told them that there were four sessions in which we aimed to train participants to improve in the game. Subsequently, we asked participants to fill out an informed consent form and a brief questionnaire including questions about age, gender, dominant hand, and possible visual impairments.

#### Baseline Session

In the baseline session, we asked participants to play the game for 15 min. We specifically instructed them to move the cursor with the stick as fast and accurately as possible between two boxes. Each time they touched a box, it turned green and participants earned a point (which would be lost again when overshooting the target). To avoid interruptions in the task performance, we instructed participants to always maintain the box-to-box movement rhythm, regardless of whether they hit or missed the box. We also emphasized that the goal of the game was to gain as many points as possible and that this could be reached with speed and accuracy.

While playing the game, participants used their non-dominant hand to move the stick, to make the game more challenging (cf. Wijnants et al., 2009, 2012). The first 30 s of the game were not measured so that participants could get used to the task, after which they performed this task for 10 min, uninterrupted. This period was chosen, because it allowed us to collect (at least) 512 data points, which is required for a reliable analysis of the complexity index (Delignières et al., 2006; see section “Complexity” for more information). When the 10 min had passed, we introduced perturbations during the last 5 min of the task. In Experiment 1, these perturbations were brief: every minute we increased the tracking speed of the cursor relative to the stick movement for 4 s, thereby interrupting the cyclic performance that participants needed to maintain. More specifically, when the tracking speed changed, identical movements of the stick temporarily resulted in faster movements of the cursor on the screen. In Experiment 2, these perturbations were prolonged: every minute we changed the tracking speed of the cursor for an entire minute, thereby forcing the participants to adapt and find another performance rhythm. In both experiments, the intensity of the change in tracking speed was different for each perturbation to make sure the participants could not get used to the changes (see Table 1).

**TABLE 1:** Schedule of tracking speed changes during the last 5 min of the task in Experiment 1 (Brief perturbation) and Experiment 2 (Prolonged perturbation).

**Brief perturbation**		**Prolonged perturbation**	
**Time (in seconds)**	**Sensitivity**	**Time (in seconds)**	**Sensitivity**
630	2	630–690	2
634	1		
690	1.5	691–750	1
694	1		
750	2.5	751–810	1.5
754	1		
810	1.25	811–870	2.5
814	1		
870	1.75	870–930	1.25
874	1		

*When the tracking speed changes from 1 to a higher number, identical movements of the stick result in faster movements of the cursor on the screen.*

#### Training Sessions

Following the baseline session, participants of Experiments 1 and 2 were involved in four training sessions, each lasting 20 min. The type of training depended on the condition—classical or differential learning—to which participants were allocated. In accordance with previous literature, at the start of each classical learning session, we provided participants with instructions on how to best execute their movements while performing the task (e.g., Schöllhorn et al., 2006, 2009, 2012). Hence, in our study we instructed participants to, amongst others, maintain a stable position; keep a delicate grip; and position the playing shoulder to the middle of the device to make sure the distance to the left and right box is equal. The remainder of each training session consisted of 10 blocks of 1.5 min each, in which the participants practiced the ideal movement, with breaks of 30 s in between the blocks. During the breaks, we gave participants corrective feedback if necessary.

In the differential learning condition, the training sessions were based on the differential learning principles outlined in previous literature (e.g., Schöllhorn et al., 2006; Savelsbergh et al., 2010; Gray, 2020). As in the classical learning condition, participants also performed 10 blocks of 1.5 min each, with breaks of 30 s in between. However, contrary to the idea of classical learning, they were not trained to perform the movement in the “right way.” Rather, in the differential learning condition we used the breaks to give participants instructions to perform additional and irrelevant movements while practicing, thereby adding noise to their movement patterns. These could, for instance, be instructions about which hand to use; sitting down or standing up; making a turn after each movement; playing with closed eyes; and standing on one leg. Additionally, we did not provide participants with any feedback on their performance or movements. This included the absence of verbal feedback, as well as feedback by the software such as earned points, elapsed time, or changing colors of the boxes when hitting or overshooting them. In the differential learning condition, each training session was different and included various instructions for the additional and irrelevant movements. Elaborate descriptions of both the classical and differential training programs are provided in Supplementary Materials.

#### Posttest

The posttest was identical to the baseline session. Hence, the participants performed the task and the first 30 s were not measured. In the next 10 min the participants played the game without being perturbed. Then, in the last 5 min the participants were exposed to either brief perturbations (Experiment 1) or prolonged perturbations (Experiment 2) with the exact same configurations as in the baseline session. At the end of the posttest, they were debriefed about the true purpose of the study.

### Measures

#### Complexity

In line with previous research on cyclic movements (e.g., walking, lateral arm movements, rowing), we used the time intervals of the left-to-right movements (between the boxes) as a unit of analysis to determine complexity. On these intervals we conducted the detrended fluctuation analysis (DFA) for the first 10 min of each participant’s baseline session (i.e., while performing the task without any perturbations). DFA is a technique to determine complexity based on temporal structures in time-series data (Peng et al., 1993). The procedure to perform the DFA was as follows. First, we turned the raw data into a time series of 512 movement intervals (from box-to-box) for each participant. These time series were then divided into bins (time scales) of equal size, in which a least-squares line was fitted to determine the trend in each bin. This trend was subsequently subtracted in the bin to detrend the time series. From the detrended time series, the root-mean-square fluctuation was calculated. This procedure was repeated using different bin sizes (i.e., 4, 8, 16, 32, 64, and 128 intervals) to identify the average fluctuation at each bin size.

**FIGURE 2:**
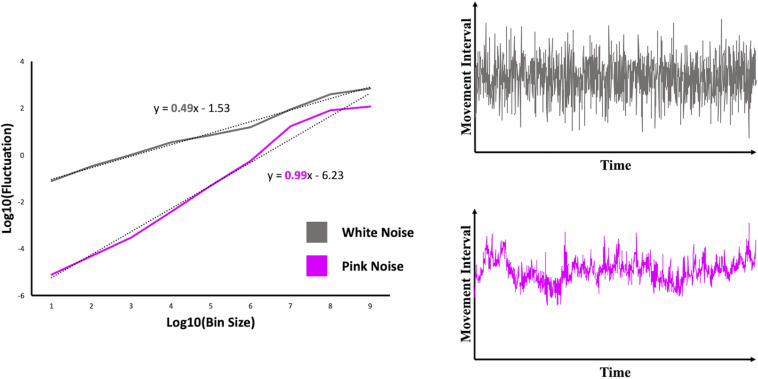
Log-log plots including a scaling relation reflecting simulated time series of white noise (gray line) and pink noise (pink line). The corresponding time series on which the scaling relations are determined, are displayed in the graphs on the right.

The relation between bin size and fluctuation reflects the DFA index (α), which corresponds to the value of the slope of a log-log plot in which fluctuation is plotted against bin size (see Figure 2). The closer the DFA index is to 1, the more it reflects a pattern of fractal scaling or pink noise in the time series. A DFA index of 0.5 reflects a white noise pattern, whereas a DFA index of 1.5 is an indication of Brownian noise (i.e., an overly regular pattern, which was not a focus in the current study).

#### Resilient Performance

In order to determine resilient motor performance of participants, we scored the number of times participants touched the boxes (without overshooting) during the periods in which they were perturbed. In Experiment 1 this score reflected the participants’ ability to maintain performance despite being briefly perturbed at regular intervals. In Experiment 2 it reflected the participants’ performance while regularly adapting to a new pattern. Indeed, each time the tracking speed of the cursor relative to the stick movements changed, the participants had to adapt to another rhythmic pattern to score points.

### Analysis

To test whether there is a positive relation between complexity and resilient motor performance (*hypothesis 1*), we determined the correlation between the DFA index and the box touches during the perturbation period in the baseline sessions of Experiments 1 and 2. To determine the effects of training on complexity (*hypothesis 2*), we conducted a repeated-measures ANOVA with DFA index (α) as the dependent variable. Session (Baseline vs. Posttest) was the within-subjects independent variable, and Training (i.e., Differential vs. Classical) and Perturbation (Brief vs. Prolonged) were the between-subjects independent variables. The latter variable allowed us to explore whether the hypothesized effect is moderated by the type of perturbation. Finally, to test the effects of training on resilient performance (*hypothesis 3*), we also performed a repeated-measures ANOVA with Box touches as the dependent variable, Session (Baseline vs. Posttest) as the within-subjects variable, and Training (i.e., Differential vs. Classical) and Perturbation (Brief vs. Prolonged) as the between-subjects variables.

## Results

### Complexity and Resilient Performance

In the first 10 min of the baseline session, we determined the DFA index and the number of box touches per minute. Taking the two experiments together, the average value of the DFA index was 0.70 (*SD* = 0.14), and the average number of box touches per minute was 69.14 (*SD* = 19.84). A Pearson correlation analysis revealed a positive correlation between the DFA index and the number of box touches in the perturbation period of the first session (*r* (78) = 0.36, *p* = 0.001, 95% CI [0.15, 0.54]). This relation was comparable between the two experiments separately (*r* (38) = 0.42, *p* = 0.008, 95% CI [0.12, 0.65] for Experiment 1, and *r* (39) = 0.32, *p* = 0.043, 95% CI [0.005, 0.58] for Experiment 2). In accordance with *hypothesis 1*, these results suggest that higher levels of complexity (i.e., α closer to 1)^1^ are related to better resilient motor performance.

### Training and Complexity

**FIGURE 3:**
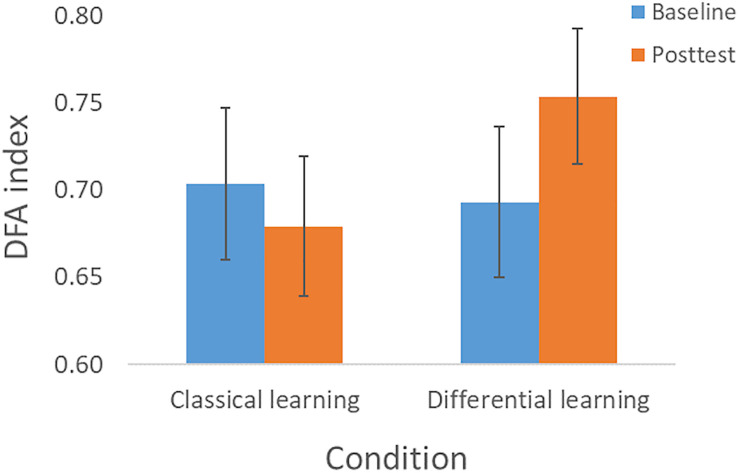
Complexity—as expressed in the DFA index—according to Session (Baseline and Posttest) and Training condition (Classical learning and Differential learning). The error bars correspond to the 95% confidence intervals.

In the baseline- and posttest we determined the complexity, and we analyzed whether changes in complexity were influenced by the type of training. The repeated measures ANOVA did not show a main effect for Session [*F*(1, 75) = 0.93, *p* = 0.34, η*_*p*_*^2^ = 0.012], nor an interaction effect of Session × Perturbation [*F*(1, 75) = 0.002, *p* = 0.96, η*_*p*_*^2^ < 0.001], or of Session × Training × Perturbation [*F*(1, 75) = 0.10, *p* = 0.75, η*_*p*_*^2^ = 0.001]. However, as expected we found an interaction effect of Session × Training with a medium effect size [*F*(1, 75) = 5.17, *p* = 0.026, η*_*p*_*^2^ = 0.065]. This effect was qualified by the finding that complexity increased in the Differential learning condition, whereas it slightly decreased in the Classical learning condition (see Figure 3). In accordance with *hypothesis 2*, the planned contrast (paired samples *t*-test) also showed a significant improvement in complexity from the Baseline session (α = 0.69, *SD* = 0.13) to the Posttest (α = 0.75, *SD* = 0.13) in the Differential learning condition: *t*(39) = −2.56, *p* = 0.014, *d* = 0.39.

### Training and Resilient Performance

In the baseline- and posttest we also determined the number of box touches per minute, and we analyzed whether changes in this number were influenced by the type of training. The repeated measures ANOVA revealed a main effect for Session with a strong effect size [*F*(1, 75) = 292.18, *p* < 0.001, η*_*p*_*^2^ = 0.80], reflecting a clear improvement in the number of box touches per minute from the Baseline session (*M* = 63.09, *SD* = 16.56) to the Posttest (*M* = 95.04, *SD* = 27.07). We also found an interaction effect of Session × Training with a strong effect size [*F*(1, 75) = 19.94, *p* < 0.001, η*_*p*_*^2^ = 0.21]. Although we detected the expected improvement in box touches from the Baseline session (*M* = 62.39, *SD* = 17.73) to the Posttest (*M* = 86.14, *SD* = 27.44) in the Differential learning condition [*t*(39) = −8.60, *p* < 0.001, *d* = 1.35], the improvement was stronger in the Classical learning condition. In the latter condition, the number of box touches per minute increased from 63.81 (*SD* = 15.47) in the Baseline session to 104.16 (*SD* = 23.70) in the posttest. In line with this interaction effect, and contrary to *hypothesis 3*, a *post hoc* independent samples *t*-test showed that the box touches were higher in the Posttest for the Classical learning condition [*t*(77) = −3.12, *p* = 0.003, *d* = 0.70].

**FIGURE 4:**
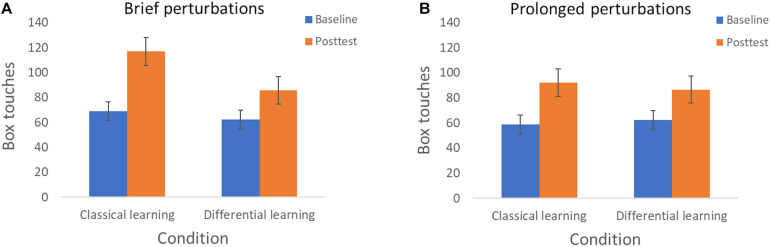
Resilient motor performance—as expressed in box touches—according to Session (Baseline and Posttest) and Training condition (Classical learning and Differential learning). **(A)** Displays the results when being exposed to brief perturbations (Experiment 1), and **(B)** displays the results when being exposed to prolonged perturbations (Experiment 2). The error bars correspond to the 95% confidence intervals.

Finally, we detected a significant Session × Training × Perturbation interaction with a medium effect size [*F*(1, 75) = 4.29, *p* = 0.042, η*_*p*_*^2^ = 0.054]. This result appears to be qualified by the observation that the beneficial effect of classical learning, in contrast to differential learning, is primarily apparent when participants are exposed to brief perturbations (Figure 4A) compared to prolonged perturbations (Figure 4B).

## Discussion

In the current research, we aimed to answer the question: What is the relation between complexity and resilient motor performance, and can they be improved through differential learning? In order to answer this question, we developed a task in which participants moved a stick from left-to-right to touch two boxes on a screen in an alternate fashion with a cursor. This task allowed us to determine the complexity of the motor performance, and to determine resilient performance by introducing perturbations while performing the task. In one of the two experiments, the emphasis was on maintaining performance while being briefly perturbed at regular intervals. In the other experiment, the focus was on prolonged perturbations that required changes to new performance states.

As expected, in both experiments we found a positive relation between complexity and resilient motor performance. Hence, whether participants needed to maintain performance while being exposed to brief perturbations (Experiment 1), or change to new performance modes (Experiment 2), higher levels of complexity were related to better performance. These results can be considered in line with Pincus and Metten (2010), who conceptualized resilience as the “metaflexibility” of a system. Their conceptualization suggests that resilience is the ability to respond to a perturbation by either becoming rigid and robust (i.e., being able to maintain a previously displayed behavioral state), or flexible and fluid (i.e., being able to easily switch to new behavioral states). Accordingly, our findings support the idea that complexity provides the system with both robustness (maintaining proper functioning despite perturbations) and adaptability (adapting to changes in the environment, see Delignières and Marmelat, 2013; Almurad et al., 2018). More generally, our results are in accordance with the consistent finding that complexity, as expressed in more prominent patterns of pink noise, is related to performance level (e.g., Wijnants et al., 2009; Den Hartigh et al., 2015; Nourrit-Lucas et al., 2015). For instance, Den Hartigh et al. (2015) found that high-skilled rowers demonstrate more prominent patterns of pink noise in their ergometer strokes than lower-skilled rowers. Furthermore, Wijnants et al. (2009) showed increased pink noise scaling when participants became trained in a simple back-and-forth movement task on a tablet. In terms of metastability, this relates to the idea that experts can adapt their movements efficiently to environmental changes (Seifert et al., 2013). More specifically, due to the abundance of motor solutions, and thereby the variability of the movements (Latash, 2012), expert motor systems may smoothly switch between different movement patterns (Kiefer et al., 2018).

In line with the interest in improving complexity (e.g., Schöllhorn et al., 2012; Almurad et al., 2018; Hill et al., 2020a), we tested whether a training program based on differential learning has, in contrast to classical learning, beneficial effects on the complexity of motor performance. Congruent with our hypothesis, we found that only differential training led to more prominent patterns of pink noise from baseline to posttest. Thus, contrary to classical learning, a differential learning program appears to increase complexity in cyclic motor performance. These results support our idea that differential learning aids in the self-organization of metastable states. Relatedly, this type of learning has previously been described as a self-organization training method (Gray, 2020).

Given the positive relation between complexity and resilient motor performance, and the beneficial effects of differential learning on complexity, a positive effect of differential learning on resilient performance was to be expected. It was, however, unanticipated that the effects of classical learning were stronger than the differential learning effects. A possible explanation for this finding is that we used a relatively simple motor task. Differential learning typically allows an individual to gain information about the entire task solution space. Tasks that require, or benefit from, the exploration of a multitude of (creative) solutions may therefore be most appropriate for a differential learning program (e.g., Santos et al., 2018). This resonates with the beneficial effects of differential learning as demonstrated in more ecological and complex tasks, such as playing soccer (Schöllhorn et al., 2006, 2012; Santos et al., 2018; Gaspar et al., 2019) speed skating (Savelsbergh et al., 2010), and playing baseball (Gray, 2020). Hence, classical training may have its benefits when there are fewer degrees of freedom in finding appropriate solutions. This idea was supported by our finding that the beneficial effects of classical training were particularly apparent in Experiment 1 that focused on maintaining the same movement pattern, compared to Experiment 2 that required finding new movement patterns every minute.

### Limitations and Future Directions

The task that we used was suitable for determining complexity based on the noise patterns in the between-box movement intervals. Indeed, much research has demonstrated that cyclical movements without external perturbations lend themselves well for the analysis of temporal structures of variation (e.g., Goldberger et al., 2002; Wijnants et al., 2009, 2012; Den Hartigh et al., 2015). However, as noted above, it is questionable whether such a task also lends itself well to reveal the possible benefits of differential learning, at least when taking resilient motor performance as an outcome variable. Future research may therefore try to extend the design of the current study to more complex cyclical movement patterns (e.g., rowing, see Den Hartigh et al., 2015, 2018). Extensions to other contexts involving more degrees of freedom are also interesting, provided that they allow the measurement of complexity and the inducement (or determination) of perturbations while performing.

Another avenue for future research is to test different self-organization training methods to improve complexity and resilience (Gray, 2020). An interesting candidate in this light is the constraints-led approach (Davids et al., 2008; Renshaw et al., 2010). In accordance with differential learning, the development of metastable states is a primary aim of this approach. However, instead of adding mere noise like in differential learning, specific task constraints are manipulated to explore the metastable region of the task space. As an illustration, a differential learning approach in boxing would consist of adding noise to the way in which an individual would punch another person, or a bag. A constraint-led approach for boxing was investigated by Hristovski et al. (2006). They manipulated for instance the distance between a boxer and another person or a punching bag. While a very close or far distance primarily affords a specific type of action (e.g., an uppercut or jab), a particular in-between distance would allow boxers to flexibly switch between actions. Training at this “edge of instability” could thereby aid in the development of adaptability of the movement system.

## Conclusion

In the current study, we aimed to connect some interesting dots on the complexity of human motor performance. Because complexity provides a system with both robustness and adaptability (Delignières and Marmelat, 2013; Almurad et al., 2018), a plausible hypothesis is that a complex system demonstrates resilience. We indeed found that individuals who revealed more prominent patterns of pink noise in their movement patterns, demonstrated better resilient performance, whether they had to adapt to brief (Experiment 1) or prolonged perturbations (Experiment 2). Furthermore, the level of complexity improved through differential learning, which is assumed to foster the development of metastable states that may improve the adaptation to perturbations. However, in our specific task classical training had more beneficial effects on resilient motor performance than differential training. To conclude, although complexity may, in general, be key for a system to adapt to stressors or perturbations while performing, an important question remains how the system can be optimally trained to respond to perturbations in different types of movement tasks.

## Data Availability Statement

The datasets and scripts used for this study can be found in the Dataverse repository: doi: 10.34894/1MXM2L.

## Ethics Statement

The protocol of the study was reviewed and approved by the Ethics Committee of Psychology, University of Groningen. The participants provided their written informed consent to participate in this study.

## Author Contributions

RD and YH developed the theoretical framework. RD, SO, and ZG prepared the experiments. SO and ZG collected the data of the experiments. RD, YH, SO, and ZG performed the data analysis, drafted the article, approved the article to be published, and agreed to be accountable for all aspects of the conducted work. All authors contributed to the article and approved the submitted version.

## Conflict of Interest

The authors declare that the research was conducted in the absence of any commercial or financial relationships that could be construed as a potential conflict of interest.

## Publisher’s Note

All claims expressed in this article are solely those of the authors and do not necessarily represent those of their affiliated organizations, or those of the publisher, the editors and the reviewers. Any product that may be evaluated in this article, or claim that may be made by its manufacturer, is not guaranteed or endorsed by the publisher.

## Footnotes

1 Values of α above 1 are also possible, and would correspond to more prominent patterns of Brownian noise. However, all of our participants demonstrated values within the range from white noise and pink noise, that is, no participant had a value of α larger than 1.
