# Supplementary material for: The Relation Between Complexity and Resilient Motor Performance and the Effects of Differential Learning
Source: Front Hum Neurosci. 2021 Aug 12;15:715375. doi: 10.3389/fnhum.2021.715375 (PMC8397476; doi:10.3389/fnhum.2021.715375)
Supplement: Supplementary file 1 [file Data_Sheet_1.docx]

**Training plan of the Classical and Differential learning conditions**

***Classical learning training plan***

During each session, the participant will train for 1.5 minutes and take breaks of 30 seconds in between. The trainer should provide feedback on the execution of the task (are they correctly following instructions?). The participant only practices with their non-dominant hand.

*Training session setup*

Software settings: 1200 seconds game; tracking speed = 1; light theme; show time and points; box width = 80; Check gain points, lose points, and blocking.

*Technique*

The device is set on a height that allows the person to hold the joystick at the exact level of their shoulder. The person should sit comfortably, with their back supported by the chair to maintain stable position. The chair should be fairly far from the table, so the participant plays with their arm mostly stretched out (at extreme points). The chair should stand a bit to the side, so that the playing shoulder is in the middle of the device. The grip should be delicate, with the weight of the hand slightly pulling the joystick towards the person.

*Trainers actions*

1. Explain the proper technique
2. Provide corrective feedback (is the participant correctly using the technique?)
3. Time the practice time / break time.

Emphasize the need to gain as many points as possible.

***Differential learning training plan***

Each of the variations listed below should be implemented for 1.5 minutes, with breaks of 30 seconds in between exercises to explain the next one (no need to switch off the game).

The trainer should not give any feedback to the participant.

Note: DH - dominant hand; NDH - non-dominant hand

*Training session 1*

Software settings: 1200 seconds game; tracking speed = 1; no feedback theme; no time indication; box width = 80.

- Standing position; NDH
- Sitting position; as far from the device as possible; NDH
- Standing; turned 90 degrees to the side; DH
- Sitting; holding the stick with two fingers, DH
- Sitting; pushing the stick with a straightened hand from side to side (no grip); NDH
- Sitting; eyes closed; NDH
- Standing; leaning forward: NDH
- Sitting; alternating hands after each move from side to side
- Sitting; gripping the joystick from the top; DH
- Sitting; gripping the joystick with two elbows

*Training session 2*

Software settings: 1200 seconds game; no feedback theme; no time indication; **inversion option turned on**; box width = 80; tracking speed follows the scheme below:

| Time | Sensitivity |
| --- | --- |
| 0 | 1.5 |
| 300 | 2 |
| 600 | 1 |
| 900 | 2.5 |

- Sitting; gripping the joystick like a pen; DH
- Standing; both hands grip the joystick
- Standing; swaying the body from left to right along with the movement of the joystick; NDH
- Sitting close to the desk; leaning back in the chair as much as possible; NDH
- Standing on one leg; DH
- Squatting; NDH
- Sitting; other arm’s elbow placed on the table; NDH
- Sitting; holding the joystick between the index and the middle finger; NDH
- Sitting; turning 360 degrees in the chair after each movement from side to side (alternating directions)
- Sitting; chair is set as low as possible; NDH

*Training session 3*

Software settings: 1200 seconds game; tracking speed = 1.5; no feedback theme; no time indication; box width = 80; **inversion option turned on**.

- Standing; one leg put forward; NDH
- Sitting; NDH moves the joystick and DH moves in the opposite direction (scissors movement) above the device
- Sitting; the other hand has to lightly tap the table while performing; NDH
- Standing; the other hand is placed on the table and acts as support because the participant is leaning forward; NDH
- Standing; walking in place while playing; NDH
- Sitting; the other hand placed on the opposite knee; DH
- Sitting; elbow raised to the level of the hand (so joystick level); NDH
- Standing; strongly gripping the joystick; NDH
- Sitting; other hand placed on the top of the head; DH
- Sitting; 45 degrees angle to the device in the opposite direction than the NDH

*Training session 4*

Software settings: 1200 seconds game; no feedback theme; no time indication; **box width 40**; tracking speed changing after the 600th second, in accordance with the following scheme:

| Time | Sensitivity |
| --- | --- |
| 0 | 1.5 |
| 600 | 1 |

- Sitting; participant should look at the joystick and not the screen; NDH
- Standing; moving from side to side with a single side step along with each movement: NDH
- Standing on widely spread legs: NDH
- Sitting; chair is set as high as possible; NDH
- Sitting; clapping after each move from side to side; DH
- Sitting as closely to the desk as possible with the device set very close to the participant; NDH
- Standing; turning the head approximately 90 degrees along with the movement of the joystick; NDH
- Sitting; the playing arm is straightened at all times; DH
- Sitting; gripping the joystick with thumb down; NDH
- Standing; pushing the joystick from side to side (only touching the joystick to initiate the movement, not at all times); hands alternate depending on the direction of the joystick
